# The pro-apoptosis effects of *Echinacea purpurea* and *Cannabis sativa* extracts in human lung cancer cells through caspase-dependent pathway

**DOI:** 10.1186/s12906-021-03204-6

**Published:** 2021-01-14

**Authors:** Fatemeh Hosami, Azadeh Manayi, Vahid Salimi, Farshad Khodakhah, Mitra Nourbakhsh, Britt Nakstad, Masoumeh Tavakoli-Yaraki

**Affiliations:** 1 Department of Biochemistry, School of Medicine, Iran University of Medical Sciences, Tehran, Iran; 2 Medicinal Plants Research Center, Faculty of Pharmacy, Tehran University of Medical Sciences, Tehran, Iran; 3 Department of Virology, School of Public Health, Tehran University of Medical Sciences, Tehran, Iran; 4 Division of Pediatric and Adolescent Medicine, Institute of Clinical Medicine, University of Oslo, Oslo, Norway; 5 Department of Pediatric and Adolescent Health, University of Botswana, Gaborone, Botswana

**Keywords:** Echinaceae purpurea, Phyto cannabinoids, *Cannabis sativa*, Cannabinoid receptor 2, Apoptosis, Cell cycle, Reactive oxygen species, Caspase 3

## Abstract

**Background:**

Considering the advantages of using medicinal herbs as supplementary treatments to sensitize conventional anti-cancer drugs, studying functional mechanisms and regulatory effects of *Echinacea purpurea* (as a non-cannabinoid plant) and *Cannabis sativa* (as a cannabinoid plant) are timely and required. The potential effects of such herbs on lung cancer cell growth, apoptosis, cell cycle distribution, cellular reactive oxygen species (ROS) level, caspase activity and their cannabinomimetic properties on the CB2 receptor are addressed in the current study.

**Methods:**

The cytotoxic effect of both herb extracts on the growth of lung cancer cells (A549) was assessed using the MTT assay. The annexin-V-FITC staining and propidium iodide (PI) staining methods were applied for the detection of apoptosis and cell cycle distribution using flow cytometry. The cellular level of ROS was measured using 7′-dichlorofluorescin diacetate (DCFH-DA) as a fluorescent probe in flow cytometry. The caspase 3 activity was assessed using a colorimetric assay Kit.

**Results:**

*Echinacea purpurea* (EP) root extract induced a considerable decrease in A549 viable cells, showing a time and dose-dependent response. The cell toxicity of EP was accompanied by induction of early apoptosis and cell accumulation at the sub G1 phase of the cell cycle. The elevation of cellular ROS level and caspase 3 activity indicate ROS-induced caspase-dependent apoptosis following the treatment of A549 cells by EP extract. The observed effects of EP extract on A549 growth and death were abrogated following blockage of CB2 using AM630, a specific antagonist of the CB2 receptor. Increasing concentrations of *Cannabis sativa* (CS) induced A549 cell death in a time-dependent manner, followed by induction of early apoptosis, cell cycle arrest at sub G1 phase, elevation of ROS level, and activation of caspase 3. The CB2 blockage caused attenuation of CS effects on A549 cell death which revealed consistency with the effects of EP extract on A549 cells.

**Conclusions:**

The pro-apoptotic effects of EP and CS extracts on A549 cells and their possible regulatory role of CB2 activity might be attributed to metabolites of both herbs. These effects deserve receiving more attention as alternative anti-cancer agents.

**Graphical abstract:**

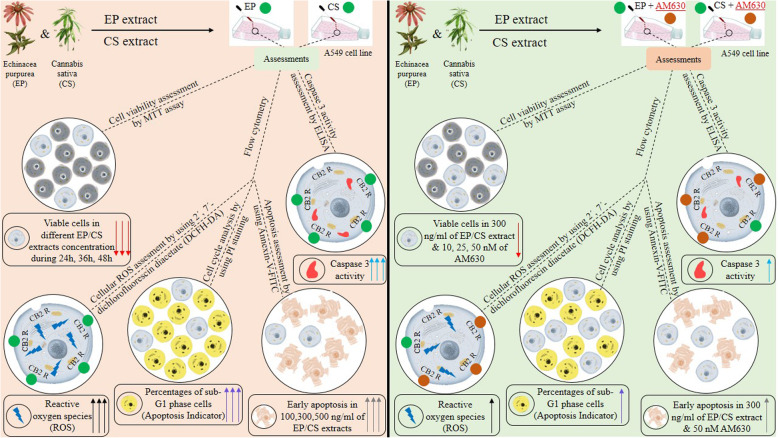

## Background

The endocannabinoid system (ECS) is an extensive lipid signaling complex system that is involved in regulating numerous physiological mechanisms and pathological processes in the human body [1]. This system consists of cannabinoid receptors, eicosanoid compounds called endogenous cannabinoids or endocannabinoids, and enzymes that synthesize and break down these compounds [2]. The cannabinoid receptors, members of the rhodopsin-like G-protein coupled receptor (GPCR) family, mediate the biological effects of cannabinoids. Cannabinoids are terpenoid lipid-based chemicals isolated from Cannabissativa (Marijuana) which has been proven to exert its effects by specific binding to cannabinoid receptors [3]. Both cannabinoid receptors and naturally occurring cannabinoids, known as phytocannabinoids, have potential therapeutic applications based on their pivotal roles in regulating immunologic responses, alleviating inflammation, tumor cell proliferation, angiogenesis, invasion, and migration [4–7].

Related to this, current antitumor agents and cytotoxic drugs are not sufficiently effective due to aberrant signal transduction and resistance in lung cancer cells, Therefore, the development of new complementary agents for the treatment of lung cancer patients is needed and necessary [8].

Interestingly, it was shown that the cannabinoid receptor 2 (CB2) was overexpressed in patients with non-small cell lung cancer (NSCLC) and tumor growth and lung metastasis was remarkably inhibited by CB2 agonists [9]. Also, it was revealed that activation of CB2 by JWH-133 inhibits epithelial to mesenchymal progression, which leads to suppression of tumor growth in NSCLC [10]. Another study [11] found that JWH-015, a CB2 agonist, could inhibit EGFR (Epidermal growth factor receptor), FAK (Focal adhesion kinase), VCAM1 (Vascular cell adhesion protein 1), and MMP2 (Matrix metallopeptidases2) pathways and prevent the accumulation of tumor-associated macrophages (TAMs) and control NSCLC lung cancer cells invasion. Also, drug-therapeutic compounds, including cannabinoids, can be transferred to lung cancer cells by inhalation with minimal side effects, using high-energy electron-dispersing nanoparticles, to induce cell death [12]. Recent studies have shown that cannabis derivatives suppress epidermal growth factor (EGF) and its downstream pathways in human lung cancer cells that cause blockage of tumor cell proliferation and migration [13]. Moreover, *Echinacea purpurea* (EP), known as coneflower, belongs to the Asteraceae family and exerts immune-modulation, anti-inflammation, anti-mutagenicity, anti-bacterial, anti-viral, larvicidal, and psychoactivity features [14]. EP can prevent virus-induced bacterial adhesion to epithelial cells and reduce the risk of respiratory inflammation and infection [15, 16]. EP modulates cytokine secretion and attenuates the clinical symptoms in mice infected by influenza [17]. Moreover, EP-isolated polysaccharides modulate the anti-tumor activity of cyclophosphamide that may be induced in mice transplanted with lung carcinoma [18]. Effects of EP can be attributed to alkamides, the major constituent of EP that have similar characteristics as cannabinoids [19, 20]. Due to this structural similarity, alkamides mimic cannabinoids and bind to cannabinoid receptors, especially CB2, then subsequently trigger the same pathways and immunologic reactions as cannabinoids do [2, 21]. Both EP root and *Cannabis sativa*, an ancient medicinal plant that contains phytocannabinoids, may play a crucial role in the pathophysiology of lung cancer, but the effects of these herbals on cancer cell growth and mechanisms of death have not been adequately addressed. In this study, we aimed to investigate the impacts of EP root and *Cannabis sativa* (CS) flower ethanolic extracts*,* on A549 lung cancer cells. We clarify and compare their possible effects on cell viability, rate of apoptosis, cell cycle distribution, level of ROSand caspase 3 activities. The CB2 antagonist AM630 was applied to delineate whether the observed effects happened in a CB2- dependent manner.

## Methods

### Chemicals and reagents

DMEM (Dulbecco’s Modified Eagle Medium) high glucose, trypsin/EDTA, NaCl/Pi, penicillin, and streptomycin were obtained from Gibco (Rockville, USA). The annexin-V-fluorescein isothiocyanate (annexin-V-FITC) apoptosis detection kit, propidium iodide (PI), 3-(4,5-dimethyltiazol-2-yl)-2,5-diphenyltetrazolium bromide (MTT), dimethyl sulfoxide (DMSO) was purchased from Sigma Aldrich (Munich, Germany). The caspase 3 colorimetric assay kit was obtained from Bio Vision (CA, USA). Fluorescent Reactive Oxygen Species (ROS) detection kit was obtained from Marker Gene Technologies (MGT, USA). The 6-iodopravadoline (AM630) was purchased from Tocris (Bristol, UK).

### EP and CS extracts preparation

The extracts of *Echinacea purpurea* (EP) root and *Cannabis sativa* (CS) female flower was prepared by the Medicinal Plants Research Center, Faculty of Pharmacy, Tehran University of Medical Sciences, Tehran, Iran. The specimens were identified by Dr. Gholam Reza Amin and deposited at the herbarium of the of Faculty of Pharmacy, Tehran University of Medical Sciences (*Cannabis sativa*: pmp-1378 and *Echinacea purpurea*: pmp-1222). Briefly, 100 g of EP roots and the flowering part of female CS flower, respectively and separately, were crushed and extracted using a maceration method (3 × 48 h). Dichloromethane was used to extract EP root and aqueous methanol (80%) applied for extraction of CS female flowers. The obtained extracts were concentrated and freeze dried to powders and kept at 4 °C until examination.

### Cell culture

The human lung cancer cell line A549 and the human normal lung fibroblast-like cell line, MRC-5 were obtained from the Pasture Institute of Iran. A549 Cells were cultured and nourished in a DMEM high glucose media supplemented with 10% fetal bovine serum (FBS) and 1% penicillin-streptomycin solution (Pen-Strep), while EMEM media supplemented with 10% fetal bovine serum (FBS) was applied for MRC-5 cell culture. Cells were maintained at 37 °C in 5% CO2 and 100% humidity. At the confluence of 70–90%, cells were collected by trypsinization and the collected cells were either used freshly, or frozen and stored at − 80 °C for subsequent experiments. Cells were seeded in 96-well plates with a density of 20,000 cells per well and incubated overnight, then the medium was replaced with fresh medium containing EP and CS extracts and different concentrations and CB2 antagonist, and AM-630 for further assays.

### Cell viability assay (MTT assay)

The cytotoxic effect of CS and EP extracts, and the agonistic effect of AM630 (6-iodopravadoline) on A549 cells were assessed using MTT colorimetric assay. Briefly, cells were cultured in 96-well plate with the density of 20,000 cells/well in DMEM high glucose, supplemented with 10% fetal bovine serum and 1% penicillin-streptomycin solution, in 5% CO2 at 37 °C. After being confluent, cells were treated with different concentrations of CS (50–900 ng/ mL) or EP (50–900 ng/ mL), respectively, and incubated for 24, 36 or 48 h. AM630 is a proven selective antagonist of CB2 receptor [22], so cells were co-treated with AM630 in a dose-dependent manner (10, 25, 50 nM) to clarify its possible effect on A549 viability. Further, we studied the effect of AM630 on the CB2 receptor, in the presence of EP or CS, respectively. Following incubation of A549 with the various extracts and/or agonist present, 20 mg/mL of 5 mg/ mL MTT solved in PBS was added to each well and incubated for an extra 4 h at 37 °C. The drug-containing medium along with MTT was then removed and replaced with a 200 mg/ mL of DMSO, which dissolved the formazan crystals and changed the color of the solution from colorless/yellow to purple. After 15 min, the plates were read with a microplate reader (Bio-Rad, CA, USA) at 570 nm and the data regarding the viability of the cells in each well was reported as the absorbance percentage of treated cells via control cells. The experiment was repeated several times and data are representative of at least three independent experiments and values are expressed in mean ± standard deviation (SD). The IC50 values were measured for EP and/or CS at each time point for both A549 and MRC-5 cells using nonlinear regression of transformed data. To determine the selectivity index (SI) which is the criterion indicating the degree of cytotoxic selectivity and specificity of compound for cancer cells against normal cells, the IC50 of EP (50–900 ng/ mL) and/or CS (50–900 ng/ mL) extracts calculated for MRC-5 cells divided by IC50 of A549 cells at each time point. The SI values more than 2 is considered as high selectivity [23].

### Apoptosis assessment

The annexin-V-FITC kit was applied to quantify the percentages of apoptotic cells using flow cytometry. Briefly, A549 cells were treated with (100, 300, or 500 ng/ mL) of CS and EP extracts, respectively, in the presence or absence of AM630 (50 nM), then harvested and washed by PBS. The cell pellets were suspended in 500 μL of 1 × binding buffer, 5 μL of annexin-V-FITC, and 5 μL of PI and incubated for 10 min at room temperature Further, samples were transferred to FACSCalibur flowcytometry (Becton Dickinson, SanJose, USA) tube and analyzed using the supplied software in the instrument.

### Cell cycle analysis

The propidium iodide (PI) staining was applied to analyze the DNA content and cell cycle distribution using flow cytometry. Cells were treated with CS (100 or 300 ng/ mL) or EP (100 or 300 ng/ mL) with and without AM630 (50 nM) for 36 h and fixed with 70% (v/v) ice-cold ethanol and washed twice with ice-cold PBS. The pellets were stained with 0.1 mg/ mL RNase A (Sigma, Munich, Germany) and 0.05 mg/ mL PI and incubated in the dark, 37 °C for 1 h. The percentages of cells in each phase of the cell cycle were determined by the FACSCalibur flow cytometer (Becton Dickinson, SanJose, USA) and the inbuilt software (BD Cell Quest software). The accumulation of cells in the subG1 phase of the cell cycle is considered as the hallmark of apoptosis.

### Measurement of intracellular reactive oxygen species (ROS)

The amount of cellular ROS is associated with the incidence of apoptosis, thus the level of ROS following treatment of cells with CS extract (100 or 300 ng/ mL) or EP (100 or 300 ng/ mL), both with and without AM630 (50 nM) for 36 h, was measured. The 2′,7′-dichlorofluorescin diacetate (DCFH-DA) as a fluorescent probe was applied for the detection of ROS level. DCFH-DA (20 μM working substrate solution) was added to the cells and incubated at 37 °C for 45 min before treatment. The level of generated fluorescence was correlated to the amount of produced ROS that was detected by FACS Calibur flow cytometer (Becton Dickinson, SanJose,USA).

### Measurement of caspase 3 activity

The level of caspase 3 activity was detected using the caspase 3 colorimetric assay Kit (Bio Vision, CA, USA). Cells were treated with CS (100 or 300 ng/ mL) or EP (100 or 300 ng/ mL) in the presence or absence of AM630 (50 nM) for 36 h. The cell pellets were lysed by a cold lysis buffer and incubated for 15 min. The reaction buffer (50 μM) contained 10 mM dithiothreitol (DTT) and 5 μM of DEVD conjugated to p-nitroaniline (DEVD-pNA) substrate (200 μM) and was added to each sample and incubated for 120 min at 37 °C. The caspase 3 activity was measured at emission wavelength 405 using a microplate reader.

### Statistical analysis

The data analysis and statistical calculations were performed by Graph Pad Prism version 6 (Graph Pad Software, San Diego, California). The non-parametric one-way analysis of variance (ANOVA) with Dennet’s and Tukey’s post hoc test was used to determine differences between groups. For determining specificity and accuracy, all experiments were implemented in triplicate and repeated at least three times. Data are presented as mean ± standard deviation (SD) and differences were taken significant for *P* < 0.05, *P* < 0.01, and *P* < 0.001. Statistical differences between different groups in each graph are marked with an asterisk. The number of asterisks indicate the degree of statistical difference and shown as * = *P* < 0.05, ** = *P* < 0.01, *** = *P* < 0.001, **** = *P* < 0.0001 in the corresponding figures.

## Results

### EP extract induced the time and dose-dependent cell death in A549 cells

**Fig. 1:**
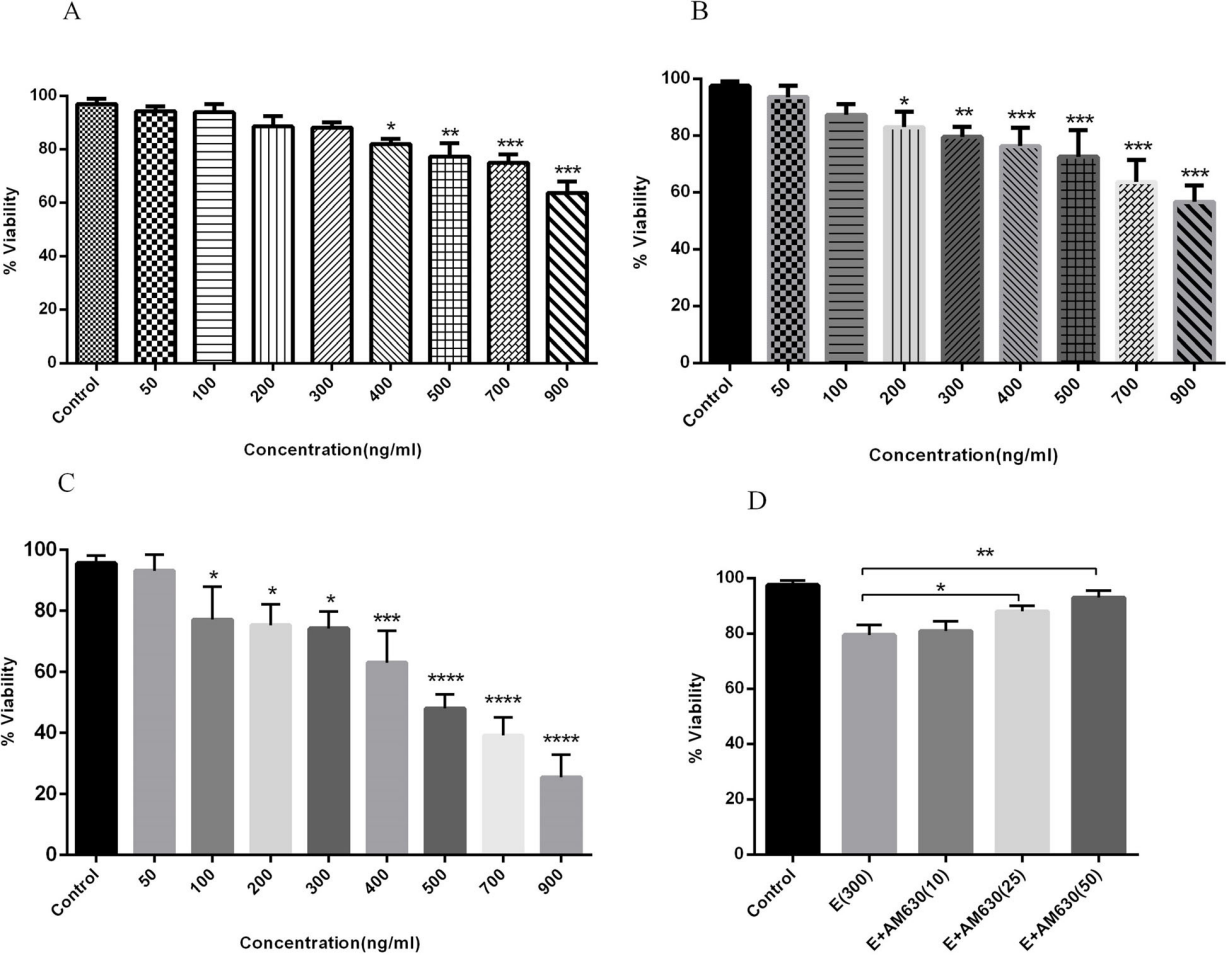
The cytotoxic effects of EP root extract on human lung cancer cells. A549 cells were exposed to different concentrations of EP extract (50–900 ng/mL) for 24 (**a**), 36 (**b**) and 48 (**c**) h. The percentages of viable cells were determined using MTT assay. Treatment with increasing concentrations of EP extract induced a time and dose dependent pattern of cell death in A549 cells. Pre-treatment with 10, 25 and 50 ng/mL of the CB2 antagonist (AM630) attenuated cell death significantly (**d**). Data represent mean ± SD of three separate experiments. The statistical differences between treated and untreated groups were analyzed by ANOVA and are indicated by asterisks (* = *P* < 0.05, ** = *P* < 0.01, *** = *P* < 0.001, **** = *P* < 0.0001)

To determine the possible effect of EP extract on human lung cancer cell growth, A549 cells were incubated with EP extract (50–900 ng/mL) for 24, 36, and 48 h, and the cell viability was measured using MTT assay. Using EP extracts, a significant reduction of viable A549 cells was observed in a dose and time-dependent pattern. (Fig. 1). After 24 h incubation, the range of 400–900 ng/mLof EP extract were able to induce cell death, effectively (Fig. 1a). Treatment of A549 cells with 300 or 500 ng/mLof EP extract for 36 h, reduced cell viability about 20 and 30%, respectively (Fig. 1b). However, the treatment of A549 cells with 300 or 500 ng/mLof EP extract for 48 h, resulted in a decrease in cell viability about 30 and 50%, respectively (Fig. 1c). The respective calculated IC50 (the half-maximal inhibitory concentration) of EP extract following 24, 36- and 48-h incubation were 1.78, 1.19, and 0.51 μg/mL. Based on data, treatment of MRC-5 cells with 50–900 ng/mL of EP extract exhibited no significant reduction in the percentages of viable cells and the maximum reduction in the cell viability percentage was less than 10% (900 ng/mL treatment for 48 h) with the IC50 value of 8.24, 5.71 and 4.62 μg/mL after 24, 36- and 48-h incubation with EP (50–900 ng/mL). The SI values calculated for EP extract was 4.61, 4.79 and 9.05 for 24, 36 and 48 h incubation, respectively which indicated the high selectivity of EP for lung cancer cells versus lung normal cells (SI > 2 indicate high selectivity). To determine the possible involvement of CB2 receptor in EP extract-induced cell death, cells were pretreated with 10, 25, and 50 nM of AM630 for 30 min before treatment with 300 ng/mLof EP extract for 36 h. It was shown that pretreatment with AM630, reverses the cytotoxic effect of EP extract on A549 cells significantly as it is shown in Fig. 1d.

### CS extract induced cell death in A549 cells in a time and dose-dependent manner

**Fig. 2:**
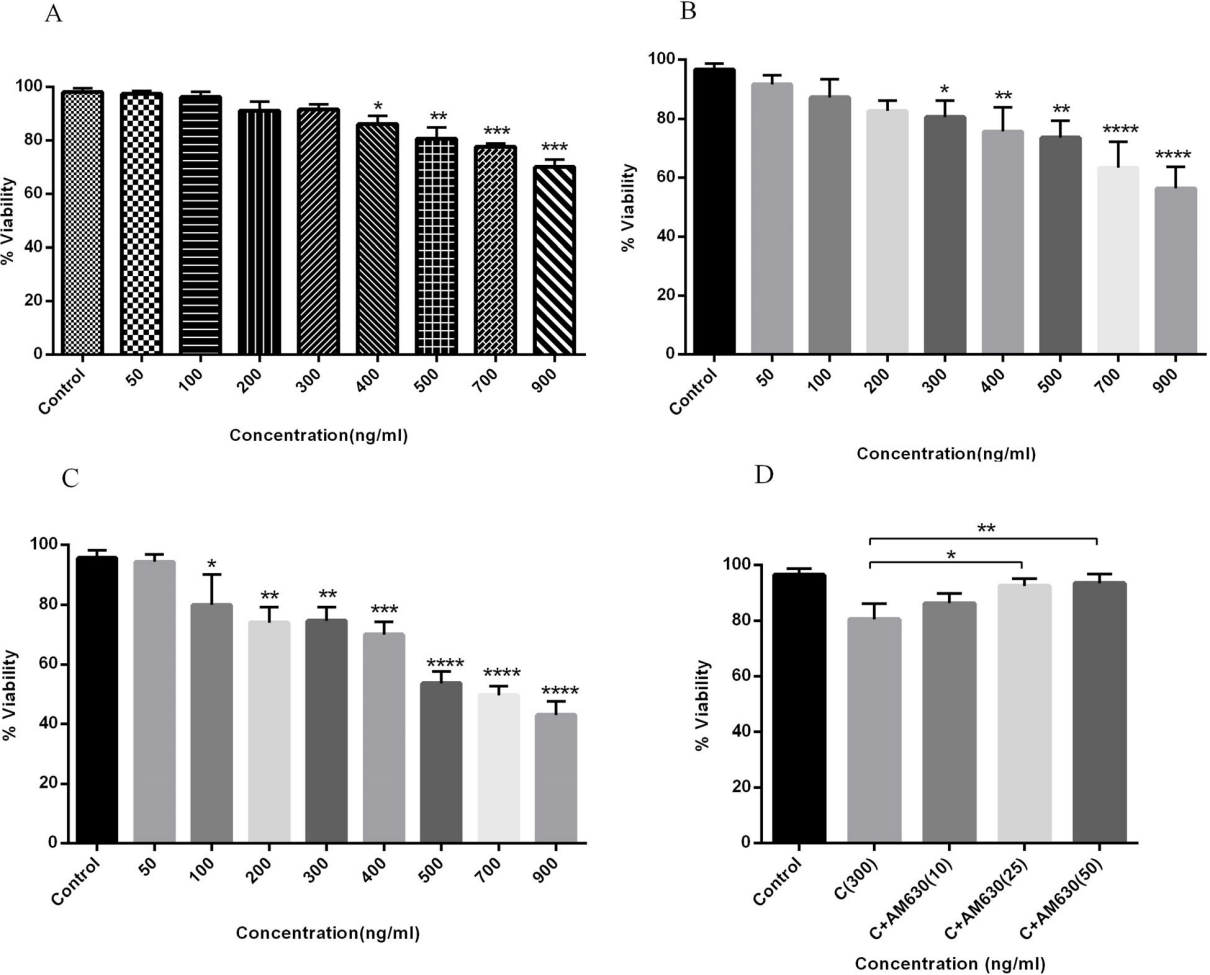
The cytotoxic effects of CS extract on human lung cancer cells. A549 cells were treated with different concentrations of CS extract (50–900 ng/mL) for 24 (**a**), 36 (**b**) and 48 (**c**) h and the percentages of viable cells were determined using MTT assay. The time and dose dependent manner of decreased cell viability is observed following CS treatment. The increasing concentrations of AM630 reduced the rate of cell death induced by CS extract (**d**). Data represent mean ± SD of three separate experiments. The statistical differences between treated and untreated groups were analyzed by ANOVA and are indicated by asterisks (* = *P* < 0.05, ** = *P* < 0.01, *** = *P* < 0.001, **** = *P* < 0.0001)

The impact of CS on the viability of human lung cancer cells was measured following treatment of cells with CS (50–900 ng/mL) for 24, 36, and 48 h. CS induced a significant reduction in the percentages of viable cells at the CS-concentrations of 400 to 900 ng/mLfollowing 24 h incubation (Fig. 2a). It was observed that after 36 h incubation, the concentrations of 300 and 500 ng/mLof CS induced a decrease in the rate of viable cells, 15 and 25%, respectively (Fig. 2b). CS-concentrations of 300 and 500 ng/mL, after 48 h incubation, induced a decrease in cell viability about 25 and 45%, respectively (Fig. 2c). The calculated IC50 of CS extract following 24, 36 and 48 h incubation was 2.28, 1.19, and 0.69 μg/mL, respectively. According to SI value calculation, our data revealed the SI values of 3.49, 4.67 and 6.75 for 24, 36 and 48 h incubation of A549 or MRC-5 cells with CS extracts, respectively. Our data revealed high selectivity of CS extract for lung cancer cells versus lung normal cells (SI > 2 indicate high selectivity). The cytotoxic effect of CS extract (50–900 ng/mL) on MRC-5 cells after 24, 36 and 48 h incubation were not significant and the maximum decrease in the percentage of viable MRC-5 cells was less than 10%. The IC50 values of 7.97, 5.56 and 4.66 μg/mL after 24, 36- and 48-h incubation of MRC-5 cells with CS (50–900 ng/mL) were obtained. We hypothesized that the CS activity was modulated through CB receptors. To clarify the role of CB2 receptor, cells were pre-treated with AM630 and incubated with CS for 36 h. We found that reduced cell viability was induced by 300 ng/mLof CS, and was abrogated following blockage of the CB2 receptor by AM 630, at 25 and 50 ng/mL (Fig. 2d).

### EP and CS extracts induced apoptosis in human lung cancer cells

**Fig. 3:**
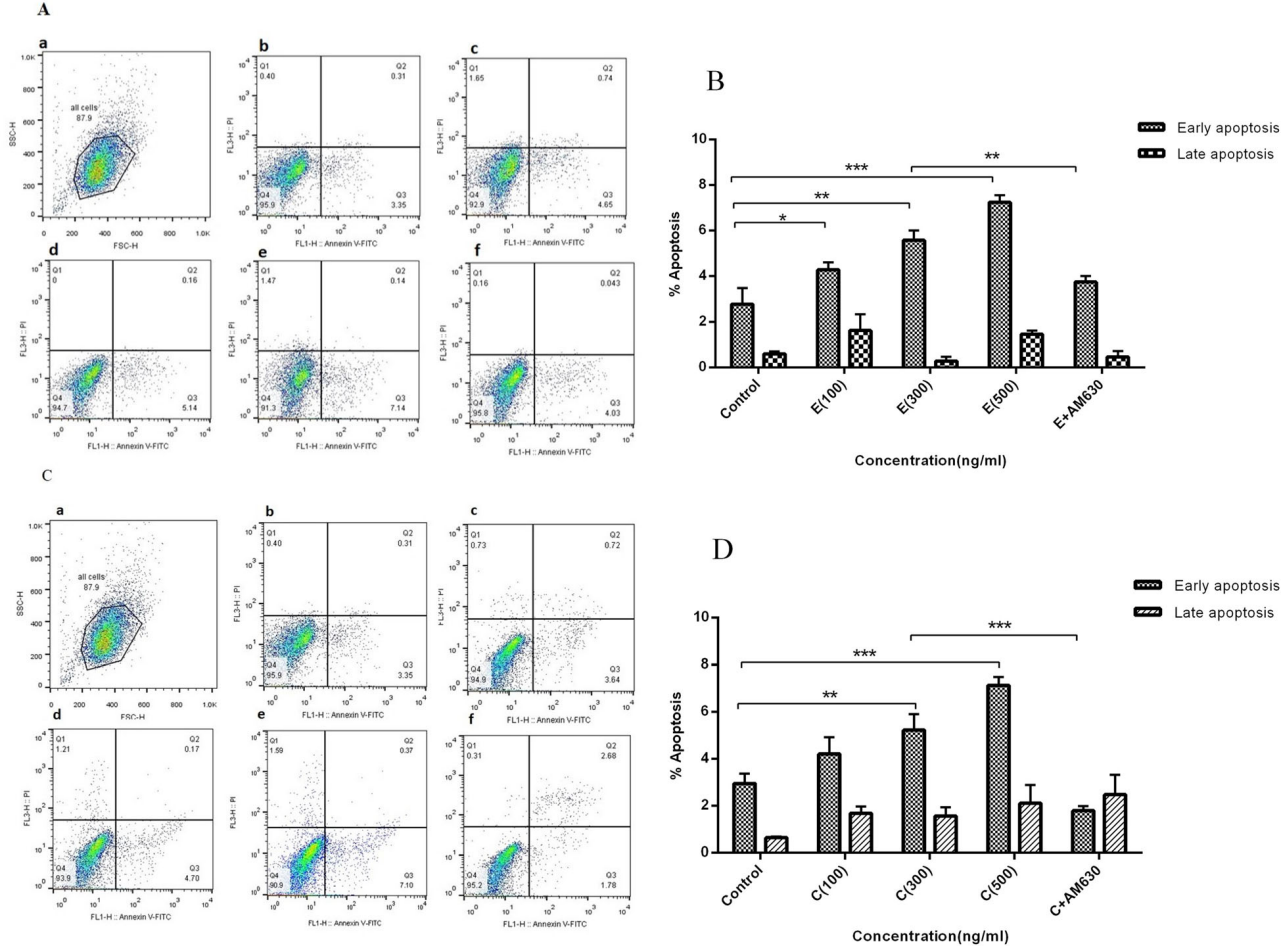
The apoptotic effects of EP and CS extracts on human lung cancer cells. The A549 cells were treated with 100, 300 and 500 ng/mL of EP and CS extracts separately for 36 h and apoptosis was analyzed by annexin-V and PI staining using flow cytometry. **A**: represents the flow cytometry histograms indicating the gating strategy (**a**), un-treated cells (**b**), cells treated with 100 ng/mL of EP (**c**), 300 ng/mL of EP (**d**), 500 ng/mL of EP (**e**) and 300 ng/mL of EP + 50 ng/mL of AM630 (**f**). **B**: represents percentages of cells at the early and late apoptosis stages following EP treatment. **C**: represents the flow cytometry histograms indicating the gating strategy (**a**), un-treated cells (**b**), cells treated with 100 ng/mL of CS (**c**), 300 ng/mL of CS (**d**), 500 ng/mL of CS (**e**) and 300 ng/mL of CS + 50 ng/mL of AM630 (**f**). **D**: represents percentages of cells at the early and late apoptosis stages following CS treatment. Data represent mean ± SD of three separate experiments. The statistical differences between treated and untreated groups were analyzed by ANOVA and are indicated by asterisks (* = *P* < 0.05, ** = *P* < 0.01, *** = *P* < 0.001)

**Fig. 4:**
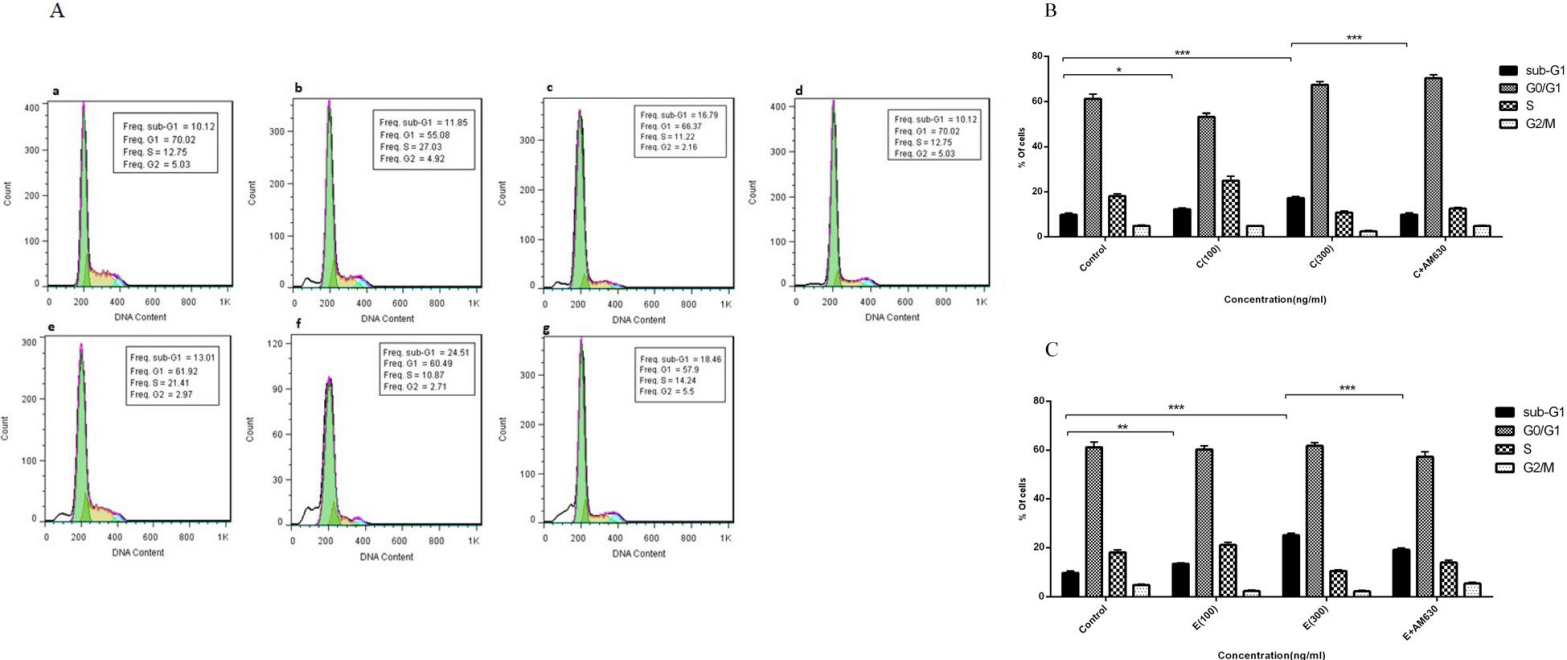
The effects of EP and CS extracts on cell cycle distribution in human lung cancer cells. The 100 and 300 ng/mL of EP and CS extracts were exposed to A549 cells for 36 h and cell cycle distribution was analyzed by flow cytometry using PI staining. **A**: represents the flow cytometry histograms indicating the un-treated cells (**a**), cells treated with 100 ng/mL of CS (**b**), 300 ng/mL of CS (**c**), 300 ng/mL of CS + 50 ng/mL of AM630 (**d**), cells treated with 100 ng/mL of EP (**e**), 300 ng/mL of EP (**f**), 300 ng/mL of EP + 50 ng/mL of AM630 (**g**) **B**: represents percentages of cells at the different phases of cell cycle following CS treatment. **C**: represents percentages of cells at the different phases of cell cycle following EP treatment. Data represent mean ± SD of three separate experiments. The statistical differences between treated and untreated groups were analyzed by ANOVA and are indicated by asterisks (* = *P* < 0.05, ** = *P* < 0.01, *** = *P* < 0.001)

To determine whether the cytotoxic effect of EP and CS on A549 cells was correlated with the induction of apoptosis, the annexin-V and PI double staining method was applied. Cells were exposed to EP extract at 100, 300, and 500 ng/mLfor 36 h, then analyzed by flow cytometry. The annexin-V positive, PI negative cells were early apoptotic cells (Fig. 3a, and c, respectively). The percentage of early apoptotic cells increased with increasing concentrations of EP extract, compared to the un-treated A549 cells (Fig. 3b), but was reduced following pre-treatment with AM630 in cell cultures of EP 300 ng/mL (*P* < 0.01). Moreover, the treatment of A549 cells with 100, 300, and 500 ng/mLof CS extract for 36 h enhanced the rate of early apoptotic cells in a dose-dependent manner compared to the un-treated A549 cells (Fig. 4d). EP at 100 ng/mLincreased the percentage of early apoptotic cells compared to the control cells, but the CS extract at 100 ng/mLfailed to induce apoptosis following 36 h incubation. Pretreatment of A549 cells with AM630 reduced the percentage of apoptotic cells exposed to CS at 300 ng/mL(*P* < 0.001) (Fig. 3d).

### EP and CS extracts induce cell cycle arrest in A549 cells

Accumulation of cells in the subG1 phase of the cell cycle, cell cycle arrest, is a hallmark of apoptosis. To study the effect of EP and CS extracts on the pattern of A549 cell cycle distribution, we incubated cells for 36 h with 100 and 300 ng/mLof EP and CS extracts, respectively, and analyzed by flow cytometry. The pattern of cell cycle distribution is shown in Fig. 4a. A549 cells accumulated in the subG1 phase following treatment with CS at 100 (*P* < 0.05) and 300 ng/mL (*P* < 0.001), compared to the untreated cells (Fig. 4b). In cell cultures exposed to CS at 300 ng/mL, pre-treatment with AM630 reduced the accumulation of cells in the subG1phase (*P* < 0.001). Exposure to resulted in increased accumulation of cells in the subG1 phase which was significant at ET 100 (*P* < 0.01) and 300 ng/mL (*P* < 0.001) compared to the control cells (Fig. 4c). The CB2 antagonist (AM630) attenuated the effect of EP at 300 ng/mLon the A549 cell cycle arrest (*P* < 0.001).

### The elevation of ROS level following EP and CS-induced apoptosis

**Fig. 5:**
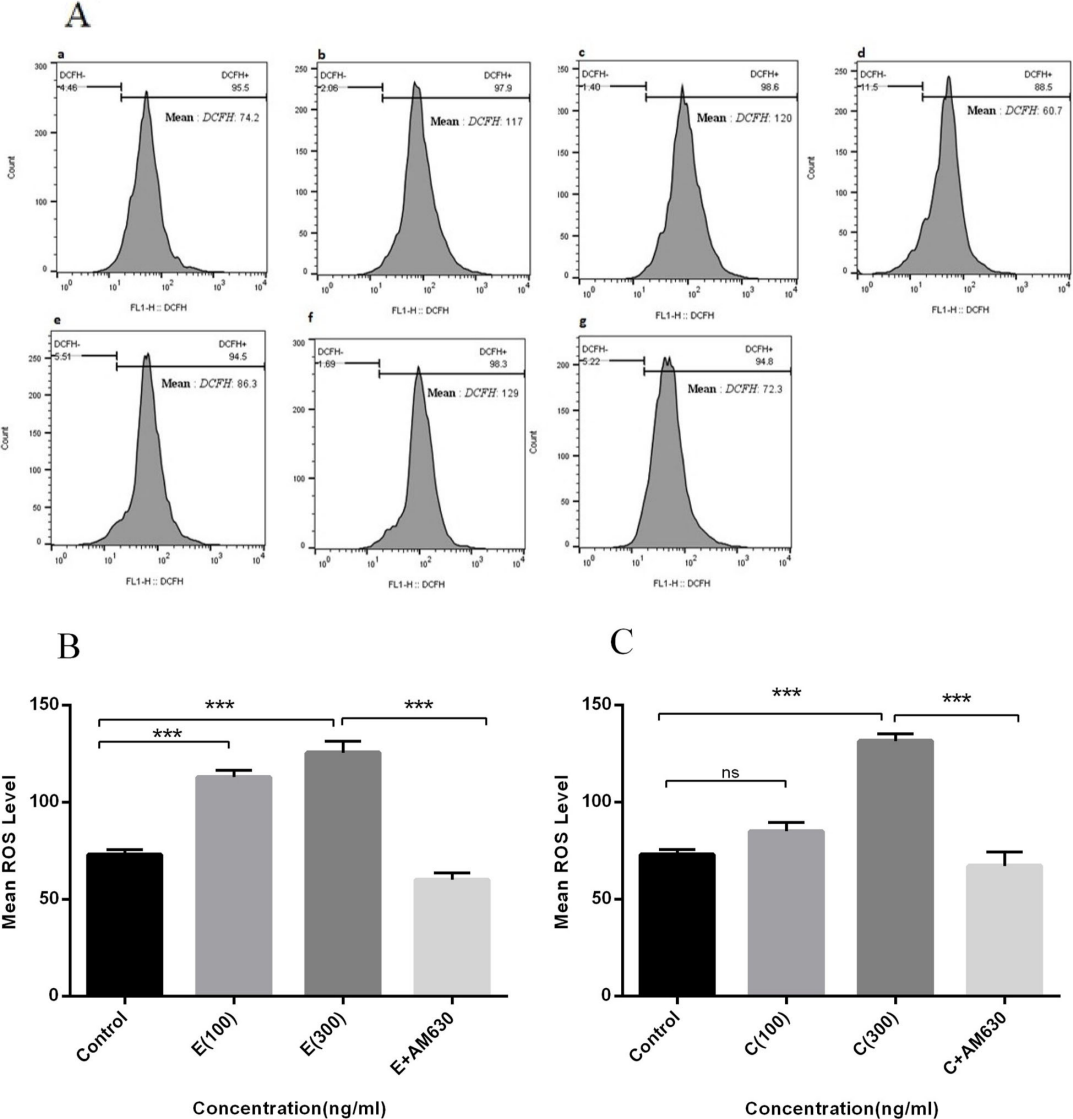
The effects of EP and CS extracts on cellular ROS level in human lung cancer cells. The A549 cells were treated with 100 and 300 ng/mL of EP and CS extracts, separately, for 36 h and subjected to ROS measurement. **A**: represents the flow cytometry histograms indicating the un-treated cells (**a**), cells treated with 100 ng/mL of EP (**b**), 300 ng/mL of EP (**c**), 300 ng/mL of EP + 50 ng/mL of AM630 (**d**), cells treated with 100 ng/mL of CS (**e**), 300 ng/mL of CS (**f**), and 300 ng/mL of CS + 50 ng/mL of AM630 (**g**). **B**: represents the mean level of ROS following EP treatment. **C**: represents the mean level of ROS following CS treatment. Data represent mean ± SD of three separate experiments. The statistical differences between treated and untreated groups were analyzed by ANOVA and are indicated by asterisks (*** = *P* < 0.001)

Elevation of cellular ROS level indicates cell stress and is accompanied with the induction of apoptosis. The cellular ROS level was measured in A549 cells following treatment with 100 and 300 ng/mLof EP and CS extract for 36 h and analyzed by flow cytometry (Fig. 5a). Data revealed an elevation in cellular ROS levels following 100 or 300 ng/mLof EP extract exposure, compared to control cells (P < 0.001) (Fig. 5b). AM630 pre-treatment attenuated this, i.e. the elevated ROS levels induced by EP at 300 ng/mL(P < 0.001) (Fig. 4b). CS at 300 ng/mLalso induced elevation of cellular ROS levels (P < 0.001), compared to the untreated cells. This was abrogated with AM630 pre-treatment (P < 0.001) (Fig. 5c). Lower CS concentrations did not induce increased ROS levels in A549 cells (Fig. 5c).

### The increased activity of caspase 3 toward EP and CS -elicited apoptosis in lung cancer cells

**Fig. 6:**
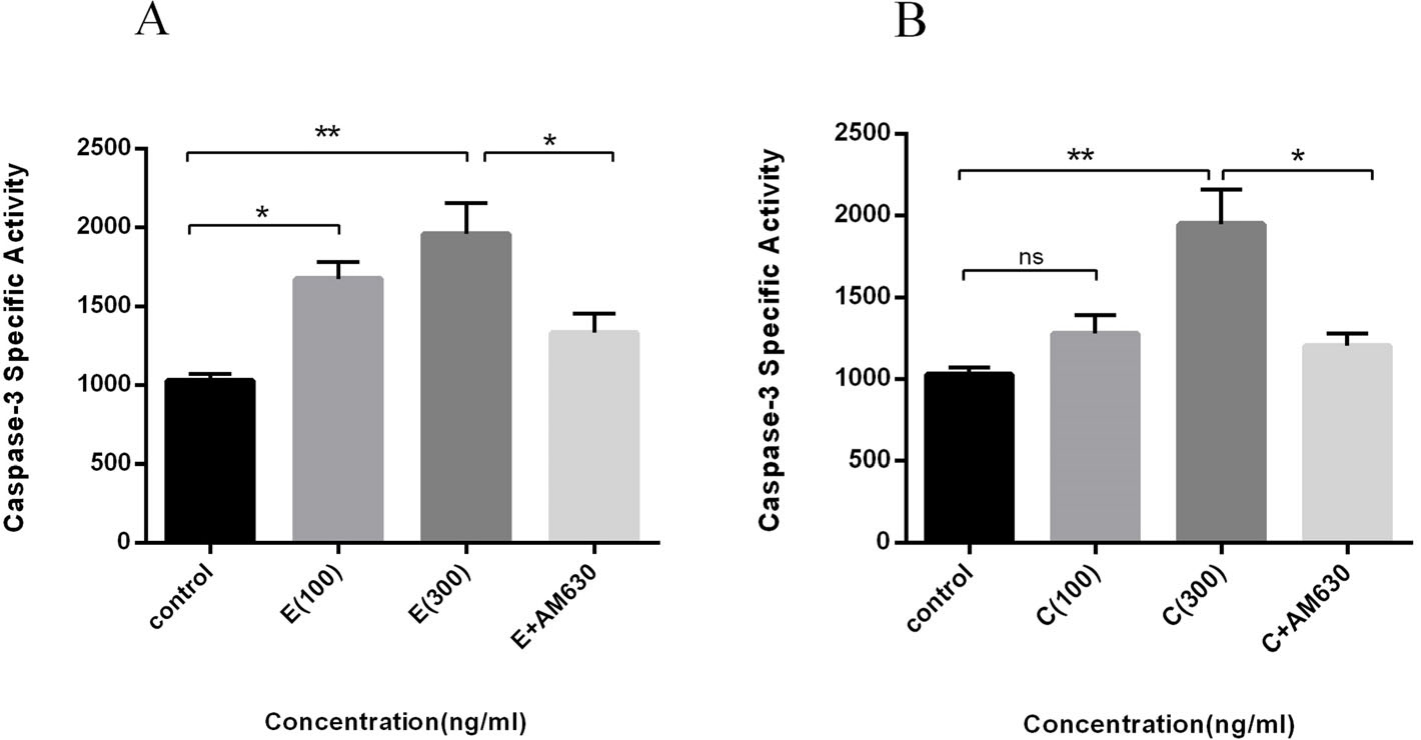
The effects of EP and CS extracts on caspase 3 activity in human lung cancer cells. Cells were treated with 100 and 300 ng/mL of EP and CS extracts for 36 h and the activity of caspase 3 measured accordingly. **a**: represents the activity of caspase 3 following 100, 300 ng/mL of EP and 300 ng/mL of EP + 50 ng/mL of AM630 treatment. **b**: represents the activity of caspase 3 following 100, 300 ng/mL of CS and 300 ng/mL of CS + 50 ng/mL of AM630 treatment. Data represent Mean ± SD of three separate experiments. The statistical differences between treated and untreated groups were analyzed by ANOVA and are indicated by asterisks (* = *P* < 0.05, ** = *P* < 0.01)

The possible involvement of caspase 3 as a mediator in apoptosis execution was assessed with increasing concentrations of EP and CS extracts. An increase in caspase 3 activity was detected with EP at 100 (*P* < 0.05) and 300 ng/mL (*P* < 0.007) (Fig. 6a), and CS extract at 300 ng/mL (*P* < 0.0073). Lower concentrations of CS did not change caspase 3 activity, compared to the untreated cells (Fig. 6b). AM630 attenuated the increased caspase 3 activity induced by EP and CS extracts (*P* < 0.05), separately.

## Discussion

Lung cancer is one of the deadliest human cancers and responsible for high rates of cancer related deaths worldwide. Effective therapeutic approaches is necessary due to its crippling effects on patient’s lives [8]. The advantages of using medicinal herbs as supplementary treatments along with the common anti-cancer treatments has received attention recently [24].

The high availability of these medicinal herbs with few side effects, and the benefit of having several treatment options make medicinal herbs useful as complementary drugs that can sensitize conventional medicines [16, 25]. *Echinacea purpurea* (EP) has attracted attention due to its wide biological effects to relieve cold symptoms, pain, seizure, wound healing, and chronic arthritis [26]. The plant derivatives have a reputation for their immune-modulating and anti-inflammatory effects since their administration enhances immune reactions and suppresses inflammation using different pathways [16, 27]. In vivo studies in mouse models showed that EP herb extract oil causes a decline in the concentration of IL-2, IL-6, and TNF-alpha and consequently suppresses inflammation [28]. EP herb extract increased the population of CD49+ and CD19+ lymphocytes in the mice spleen, increased the cytotoxic activity of natural killer (NK) cells and the level of interferon-alpha inhibited release of tumor necrosis factor-γ and IL1β and ultimately improved both innate and adaptive immune responses [29]. Additionally, its cytotoxicity and anti-tumorigenic features provided interesting evidence, but documentation of a direct inhibitory effect of EP derivatives on cancer cell growth is lacking. On the other hand, the remarkable anti-inflammatory capability of EP derivatives along with their immune-enhancing features, indicate an efficient alternative for suppressing tumor growth, stimulating the immune system, and creating a barrier against tumor cells. In support of this, this study applied an appropriate range of EP extract concentrations (50–900 ng/mL), at different time intervals, to clarify the possible cytotoxic effect of EP on lung cancer cells. Our data revealed that EP extract reduced the number of viable lung cancer cells in a time and dose-dependent manner. The cytotoxic effect was accompanied by the induction of apoptosis in the A549 cells. To the best of our knowledge, the relevance of EP extracts in the treatment of the lung cancer has not been investigated in previous studies, although daily administration of EP results in an increase in NK cells in the human bone marrow, generating sites and stimulation of NK cell activity, thereby improving the functionality of the immune system that may fight cancer cells [30]. EP flower extract and its major derivative, cichoric acid, induced apoptosis and inhibited tumor growth via inhibition of telomerase activity in human colon cancer cells, Caco-2 and HCT-116 cells [31]. Further, EP root extract induced apoptosis via DNA fragmentation and increased of caspase 3/7 activity in a dose and time dependent manner in human pancreatic cancer cells, MIA PaCa-2, and colon cancer cells, COLO320 [32]. Our data indicate that EP root extract induces accumulation of A549 cells in the subG1 phase, in a dose-dependent manner which accounts as a hallmark of apoptosis. Caspase activation is required for the execution of apoptotic cell death, but apoptosis may also be induced through a caspase-independent manner [33]. In accordance, the generation of cellular ROS, as inevitable byproducts of metabolism, is related to the induction of caspase activity and mediates apoptotic cell death [34]. Based on our data, exposure of A549 cells by increasing concentrations of EP root extract resulted in elevation of cellular ROS. EP extract administration caused an increase in the caspase 3 activity, indicating induction of caspase-dependent cell death following EP extract treatment. EP secondary metabolites such as alkamides are likely to be responsible for these pharmacological effects as they share structural similarity with cannabinoids components like anandamide [16].

Cannabinoids refer to a group of components with diverse biological effects that are mediated through activation of their specific G-protein-coupled receptors, namely, CB1 and CB2 [4, 35, 36]. Several cancer-related properties are attributed to the cannabinoids and their receptors, including tumor growth suppression, apoptosis induction, angiogenesis inhibition, and manipulation cancer related signaling pathways [6, 7]. Cannabis derivatives and CB receptor activation has been reported to inhibit tumor growth through a wide range of mechanisms and in various types of tumors including non-small lung cancer [9], prostate cancer [5], breast cancer [7], mantle cell lymphoma [37], glioma [38], hepatocarcinoma [39] and colon cancer [40]. The present study revealed that the CS extract induced lung cancer cell death in a dose and time-dependent manner. The cytotoxic effect on A549 cells was accompanied by the induction of apoptosis and accumulation of cells in the subG1 phase of the cell cycle. In line with this finding, a cytotoxic effect of cannabidiol (CBD), the major derivative of Cannabis, was observed in the MDA-MB-231 breast cancer cell line and colon cancer cells [7, 31]. In the current study, the increased activity of caspase 3 and concurrent elevation of cellular ROS level following CS extract administration, indicate the possible involvement of ROS in the cannabis-induced caspase-dependent apoptosis in lung cancer cells. It was shown that, WIN-55 as a CB receptor agonist induced accumulation of cells in the sub G1 stage and overstimulation of caspase 3/7 in PC-3 prostate cancer cell lines [37]. Previously reported results were consistent with our observations, and showed an enhancement in the level of ROS in breast cancer cells which were induced by Cannabidiol (CBD) treatment [41]. Constituents of EP and CS herb plants can stimulate cannabinoid receptors and subsequently trigger the same pathways and immunologic reactions. Therefore, we assessed the possible role of the CB2 receptor in the apoptosis-induced effects induced by EP and CS extracts. M630, a CB2 antagonist, attenuated the rate of cell death, apoptosis, accumulation of cells in the sub G1 phase, ROS level, and caspase 3 activity, all induced by both EP and CS extracts in A549 cells. Based on these observations, the metabolites of EP (alkamides) and CS cannabinoids might mediate their effects through the CB2 receptor in A549 cells. In support of this, binding of alkamide to the CB2 receptor is required for the subsequent anti-inflammatory impact of alkamide [42]. Additionally, it was shown that the extracted alkamides from EP root stimulate IL-10 and inhibit tumor necrosis factor (TNF), and ultimately carry out the immunomodulatory effects and suppress inflammation [43]. In conclusion, the pro-apoptotic impact of EP and CS extracts on lung cancer cells and the modulating involvement of the CB2 receptor was evaluated. The cannabinomimetic properties of EP root extract on the CB2 receptor might be attributed to the alkamides. These favorable effects of the EP root extract on cell death, apoptosis, cell cycle distribution, ROS level, and caspase activity elucidated consistency with the CS mentioned effects on the A549 cell line.

## Conclusion

Based on the findings, it can be postulated that EP and CS extracts can inhibit lung cancer cell growth and induce apoptosis and should be considered as an alternative anti-cancer agent in lung cancer. However, more research is required to elucidate the detailed underlying molecular mechanism of action and the exact role of each derivative of these herbal extracts. Therefore, fractionation of both herbal extracts to purify active compounds and investigating the molecular pathways influenced by are highly recommended.

## Data Availability

The data used to support the findings of this study are included within the article and any further information can be provided by corresponding author upon request.

## Abbreviations

ECS: Endocannabinoid system
CS: *Cannabis sativa*
EGFR: Epidermal growth factor receptor
FAK: Focal adhesion kinase
VCAM1: Vascular cell adhesion protein 1
MMP2: Matrix metallopeptidases2
TAMs: Tumor-associated macrophages
EGF: Epidermal growth factor
EP: Echinaceae purpurea
NKs: Natural killer cells
PMN: Poly morpho-nuclear leukocytes
CB2: Cannabinoid receptor2
GPCR: G-protein coupled receptor
NSCLC: Non-small cell lung cancer
ROS: Reactive oxygen species
DMEM: Dulbecco’s Modified Eagle Medium
EMEM: Eagle’s Minimum Essential Medium
MTT: 3-(4,5-dimethyltiazol-2-yl)-2,5-diphenyltetrazolium bromide
DMSO: Dimethyl sulfoxide
FBS: Fetal bovine serum
Pen-Strep: Penicillin-streptomycin solution
DCFH-DA: 2′, 7′-dichlorofluorescin diacetate
ANOVA: Non-parametric one-way analysis of variance
NK: Natural killer
IL: Interleukin
CBD: Cannabidiol
PI: Propidium iodide
DTT: Dithiothreitol
DEVD-pNA: DEVD conjugated to p-nitroaniline
Annexin-V-FITC: Annexin-V-fluorescein isothiocyanate
